# Comparison of the Effects of Feeding Compound Probiotics and Antibiotics on Growth Performance, Gut Microbiota, and Small Intestine Morphology in Yellow-Feather Broilers

**DOI:** 10.3390/microorganisms11092308

**Published:** 2023-09-13

**Authors:** Yuyan Feng, Xiaoting Wu, Dan Hu, Canyang Wang, Qu Chen, Yingdong Ni

**Affiliations:** 1 Key Laboratory of Animal Physiology & Biochemistry, Nanjing Agricultural University, Nanjing 210095, China; 2 State Key Laboratory for Managing Biotic and Chemical Threats to the Quality and Safety of Agro-Products, Institute of Agro-Product Safety and Nutrition, Zhejiang Academy of Agricultural Sciences, Hangzhou 310021, China

**Keywords:** probiotics, antibiotics, gut microbiota, yellow-feather broilers

## Abstract

This study was devoted to the comparison of the probiotic effect of compound probiotics to antibiotics as a feed additive for chicken. Two hundred and seventy newly hatched yellow-feather broilers were randomly divided into three groups: the control group (Con), probiotics (Pb), and antibiotics group (Ab). The Pb group received compound probiotics (*Bifidobacterium*, *Lactobacillus acidophilus*, *Streptococcus faecalis*, and yeast) via drinking water for 24 days. The Ab group received antibiotics (zinc bacitracin and colistin sulfate) in their diet for 24 days. All broilers were slaughtered on day 42. Compared with the Con group, the body weight was significantly increased on days 13, 28, and 42 in the Pb group (*p* < 0.05), and markedly increased on day 28 in the Ab group (*p* < 0.05). Compared with the Ab group, the body weight of the broilers in the Pb group increased significantly on day 13 (*p* < 0.05). Compared to the Con and Pb groups, the antibiotics treatment reduced the feed intake (*p* < 0.05), but there was no significant difference in the feed conversion ratio between the Ab and Pb groups (*p* > 0.05). The feed conversion ratio of the broilers treated with antibiotics or probiotics significantly decreased compared to the Con group (*p* < 0.05). The depth of duodenum, jejunum, and ileum crypts in the Pb group decreased significantly compared to the Con and Ab group (*p* < 0.05). The ratio of the villi length to crypt depth of duodenum, jejunum, and ileum epithelium was significantly increased in the Pb group compared to the Con group (*p* < 0.05). The genera *Bacteroides* and *Barnesiella* were the most significantly enriched bacteria in the Ab and Pb groups, respectively (*p* < 0.05). The expression of the genes related to antibiotic resistance was significantly decreased in the Pb group compared to the Ab group (*p* < 0.05). Although both compound probiotics and antibiotics can improve growth performance, antibiotics increased the abundance of harmful bacteria and drug-resistant genes, while probiotics increased *Barnesiella* abundance, which is related to a decrease in the drug-resistant gene expression. Moreover, the probiotics treatment improved small intestinal morphology and fecal emissions, while antibiotics have no significant effect on these indicators, indicating a bright future for probiotics as an alternative to feed antibiotics in the yellow-feather broiler industry.

## 1. Introduction

Antibiotic abuse leads to the prevalence of resistant bacteria, which pose a serious threat to animal welfare, human health, and the environment [1,2]. The use of antibiotics in animal feeds has been completely banned in China from 1 July 2020. Therefore, there is an urgent need to find alternatives to antibiotics for improving welfare and growing performance in the poultry industry. In recent years, probiotics have been considered a possible alternative to antibiotics in animal feed [3].

Probiotics, as a potentially green alternative to antibiotics, have attracted a lot of attention due to their beneficial effects on humans and animals in recent years. Supplementing animal diets with probiotics can improve feed efficiency and growth performance [4,5]. Zeng et al. reported that supplementation with compound probiotics based on *Bacillus spp.* and *Clostridium butyricum* can improve the immune function, growth performance, and morphology of the small intestine [6]. The components of compound probiotics were diverse in different studies. In this study, the compound probiotics were mainly based on live *Bifidobacterium*, *Lactobacillus acidophilus*, *Streptococcus faecalis*, and yeast preparation. *Lactobacillus* and *Bifidobacterium* are the most widely used as alternative antibiotics in the poultry industry. A previous study showed that dietary supplementation with *Lactobacillus acidophilus* not only improved growth performance, but also reduced the mortality rate of broilers caused by *Escherichia coli* [7]. Several strains of *Bifidobacteria* have been used in modulating the gut microbiome; for example, it has been shown that *Bifidobacteria bifidum* and *Bifidobacteria longum* can inhibit a wide range of gram-negative and gram-positive bacteria through producing antimicrobial substances [8,9]. *Streptococcus faecalis* has been reported to possess adhesion abilities, produce bacteriocins (antimicrobial peptides, AMPs), and provide immunostimulation [10], thus improving the homeostasis of the gut microbiota [11]. Additionally, feed supplementation with live yeast can improve gut histological morphology, inhibit pathogens colonization, and enhance nutrient digestion and absorption [12].

At present, there are still some limitations in the research of probiotics in broilers; on the one hand, most of the studies focused on the impact of a single strain of probiotic on chicken welfare and growth performance, and there is a lack of comprehensive evaluation of compound probiotics. On the other hand, the research is mainly concentrated on white-feather broilers. However, the dietary nutritional composition and genetic background are different between yellow-feather broilers and white-feather broilers, which are the important factors affecting the gut microbiota. Therefore, the aim of this present study was to undertake a comprehensive characterization of growth performance, blood parameters, gut microbiota, intestinal epithelial structure, microbiota antibiotic resistance, and excreta nitrogen and phosphorus emissions of yellow-feather broilers fed with compound probiotics in comparison with the addition of antibiotics.

## 2. Materials and Methods

### 2.1. Ethics Statement

Animal procedures were permitted by the Institutional Animal Care and Use Committee (IACUC) (njau-2020-009) of Nanjing Agricultural University according to the Guidelines for Experimental Animals of the Ministry of Science and Technology (2006, Beijing, China).

### 2.2. Experimental Design and Sampling

A total of 270 one-day-old yellow-feather chickens were purchased from Jiangsu Huaxigen Company (Nanjing, Jiangsu, China). Broilers were randomly allocated to experimental cages in 6 replicates of 15 broilers. All chicks were exposed to continuous illumination and free access to food and water. The ambient temperature was maintained at 35 °C~37 °C during the first 3 days and then was gradually decreased by 0.5 °C every day until reaching a final temperature of 21 °C. The basal diet in 2 phases (1~21 d and 22~42 d) was formulated to meet the specifications for yellow-feather broilers suggested by the NRC (NRC, 1994) [13] and Nutrient Requirements of Yellow-Feathered Broiler (NY/T 33, 2004, China). In addition, the basal diet contained no antibiotics (Table 1).

Broilers were randomly divided into the control (Con) group, probiotic (Pb) group, and antibiotic (Ab) group and allocated to experimental cages in 6 replicates of 15 broilers. After a prefeeding period of 2 days, Pb group chickens received 100 mg probiotic mixtures per chicken via drinking water for 24 days. Compound probiotics provided by a commercial company (Jiangsu H.F.Q. Technology Co., Ltd., Nantong, Jiangsu, China) contained *Bifidobacterium*, *Lactobacillus acidophilus*, *Streptococcus faecalis*, and yeast. *Bifidobacterium* and *Lactobacillus acidophilus* were not less than 1.0 × 10^7^ CFU, and *Streptococcus faecalis* and yeast were not less than 1.0 × 10^6^ CFU per gram. These strains were isolated from the gut microbiota of chickens and cultured in commercial fermentation tanks. Ab group chickens were added antibiotics (16.5 mg/kg zinc bacitracin + 3.3 mg/kg colistin sulfate) in their diet for 24 days.

After 12 h of feed withdrawal, 2 birds close to average body weight per replicate were weighed and then electrically stunned and exsanguinated on D21 and D42, respectively. Blood samples were collected from the jugular vein into 5 mL heparinized tubes, and plasma was then obtained after centrifuging at 2000× *g* for 10 min at 4 °C. The plasma samples were stored at −20 °C for further analysis of biochemical parameters. After opening lengthwise, small intestinal tissues were rinsed with saline and fixed with 4% paraformaldehyde. The cecal contents were collected into a 10 mL sterile tube, quickly frozen with liquid nitrogen, then stored at −80 °C for 16S rRNA sequencing. The liver, breast muscle, abdominal fat, and bursa of Fabricius samples were collected and weighed individually to measure their organ indexes.

### 2.3. Measurement of Growth Performance

Birds were weighed at D3, 13, 28, and 42 on a per pen basis. Feed intake was recorded daily on a per-replicate basis. A daily mortality check was conducted, and dead birds were weighed to adjust estimates of gain, intake, and feed conversion ratio. The final body weight, average daily feed intake, average daily gain, and feed conversion ratio were calculated.

### 2.4. HE Staining and Histomorphology Analysis

Small intestinal tissues were collected and soaked in 4% paraformaldehyde immediately. The fixed intestinal samples were dehydrated, embedded in paraffin wax, and cut into serial sections (4–5 μm). The sections were dewaxed, then rinsed with alcohol and distilled water, stained with hematoxylin, rinsed with tap water and 1% hydrochloric acid ethanol, then stained with 5% eosin. Small intestine tissue sections were evaluated by a microscope coupled with a Microcomp integrated digital imaging analysis system (Olympus SZX10, Tokyo, Japan). Three orientated sections cutting vertically from the villus enterocytes to the muscularis mucosa were selected from each sample and the measurements were carried out following the previous study [14]. Statistical significance of the results was tested using one-way ANOVA (version 20.0, SPSS Inc., Chicago, IL, USA), and statistical significance was determined at *p* < 0.05.

### 2.5. Extraction of Genomic DNA and 16S rRNA Sequencing

Total genomic DNA from samples was extracted using the cetyl trimethylammonium bromide/sodium dodecyl sulfate (CTAB/SDS) method. DNA concentration and purity were monitored on 1% agarose gel. The 16S rRNA genes in distinct regions (16S V3-V4) were amplified using specific primers with barcodes. Phusion^®^ High-Fidelity PCR Master Mix with GC buffer from New England Biolabs and high-fidelity enzyme were used for PCR to ensure the efficiency and accuracy of amplification. PCR product was verified by electrophoresis on a 2% agarose gel, then recycled by using GeneJETTM Gel Extraction Kit (Thermo Scientific, Waltham, MA, USA). The sequencing libraries were performed using Ion Plus Fragment Library Kit (Thermo Scientific, MA, USA), and then they were sequenced on an Ion S5^TM^ XL platform. Uparse software was used to perform sequence analysis. Species annotation analysis was measured using the Mothur method and the Silva Database. The data were normalized using a standard of sequence numbers according to the least sequences.

### 2.6. Processing of Sequencing Data and Functional Prediction

Sequence analysis was performed by using Uparse software package (Uparse v7.0.1001, http://www.drive5.com/uparse/, accessed on 9 January 2022) [15]. Alpha diversity within samples and beta diversity among samples were analyzed by using in-house Perl scripts and visualized by R software (Version 2.15.3). Sequences with ≥97% similarity were assigned to the same operational taxonomic units (OTUs). The species annotation of OTUs was performed using MOTHUR (http://www.mothur.org, accessed on 12 January 2022) and SSUrRNA database in SILVA138 (http://www.arb-silva.de/, accessed on 15 January 2022) [16]. Spearman correlation analysis between microbiota and environmental factors was performed using corr.test function in psych package, and the pheatmap function was used for visualization. Functional capacity of the cecal microbial community was predicted by using the Tax4Fun software. The significant differences in KEGG (Kyoto Encyclopedia of Genes and Genomes) abundances between groups were identified and analyzed by using the KEGG Mapper pathway search function.

### 2.7. Quantification of Antibiotic Resistance Genes

Real-time quantification PCR (qPCR) was used to quantify the abundance of the antibiotic resistance genes. The primers used for qPCR are listed in Table 2. The Ct value was used to calculate the number of corresponding gene copies according to the standard curves. Three replicates of each sample were tested by qPCR reaction. The concentrations of each DNA sample were adjusted to 10 ng/uL. The qPCR mixtures consisted of 0.2 μM concentration of each primer, 10 μL of Genious 2X SYBR Green SYBR qPCR mix (ABclonal, Wuhan, China), 1 μL of template DNA, and the final volume was adjusted to 25 mL by adding DNase-free water. Mx3000 P (Stratagene, San Diego, CA, USA) was employed for amplification and quantification. The protocol was conducted as follows: 30 s at 95 °C, then 40 cycles of 5 s at 95 °C, 30 s at the annealing temperature, extension for another 30 s at 72 °C and a simultaneous fluorescence signal scanning at 72 °C, and then a melt curve stage with temperature ramping from 65 to 95 °C. The relative expression level was calculated using the 2^−ΔΔCt^ method.

### 2.8. Plasma Biochemical Analysis

Plasma samples were measured by 7020 automatic biochemical analyzer (HITACHI, Tokyo, Japan) for determination of glucose, cholesterol (CHOL), high-density lipoprotein (HDL), low-density lipoprotein (LDL), aspartate transaminase glucose (AST), alanine transaminase (ALT), triglyceride (TG), and albumin (ALB) concentrations.

### 2.9. Determination of Total Phosphorus, Nitrogen, and Ammonia Nitrogen in Feces

Fecal samples were collected from each pen for statistics of average excretion on D42. The fecal samples were put into polyethylene self-sealing bags and air-dried in the laboratory. After grinding and sieving, the fecal samples were used for analysis. After digestion with H_2_SO_4_-H_2_O_2_, the total nitrogen content of fecal samples was determined by automatic Kjeldahl apparatus (KETUO, KDY-9820, China), and the total phosphorus content of fecal samples was determined using anti-molybdenum-antimony colorimetry. The fecal samples used for ammonia nitrogen detection were first extracted with 1 mol/L KCl, and then the ammonia nitrogen content of extracting solution was determined using indophenol blue method. Ultraviolet spectrophotometer (Shimadzu, UVmini-1240, Japan) was used for the detection of total phosphorus and ammonia nitrogen.

### 2.10. Statistical Analysis

Differences in gut microbiota composition and function were analyzed using Wilcoxon (two groups) or Kruskal–Wallis (three groups) tests, and the *p*-value was adjusted by Benjamini–Hochberg correction. The result of LEfSe difference analysis (a threshold value of >3 means, *p* < 0.05) was filtered by using Kruskal–Wallis test. The other data, like growth performance, statistics of morphology, gene expression, nitrogen, and phosphorus emissions, were subjected to statistical analysis by one-way ANOVA using the SPSS software (version 20.0, SPSS Inc., Chicago, IL, USA). Significant differences between means were compared using Duncan’s multiple range test. The replicate (*n* = 6) was considered as the experimental unit. The correlations between cecal microbiota and antibiotic-resistant genes were assessed by Spearman’s correlation analysis. The results are presented as the mean ± standard error. Statistical significance was determined at *p* < 0.05.

## 3. Result

### 3.1. Body Weight, Food Intake, and Feed Conversion Ratio

**Figure 1:**
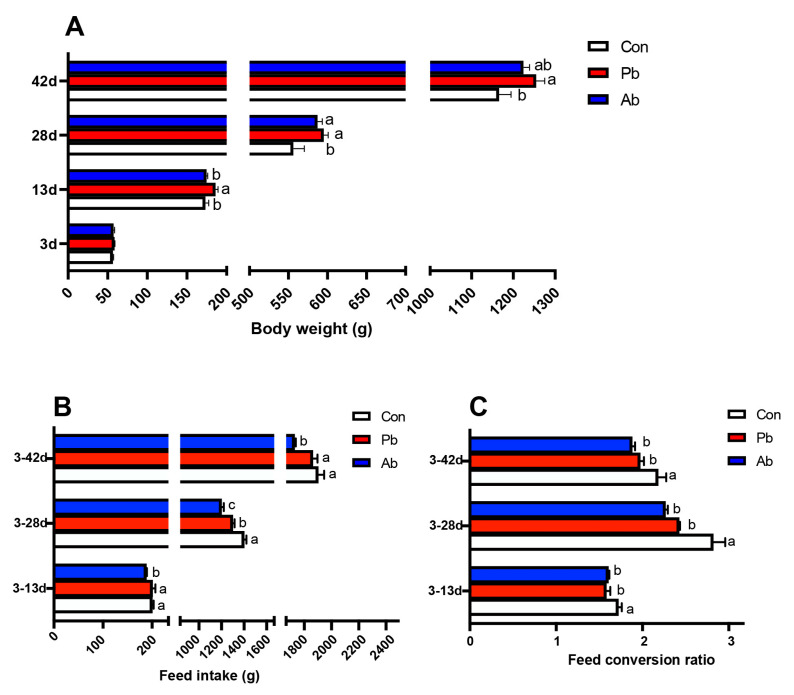
The effects of antibiotics and compound probiotics on growth performance. (**A**) Weight statistics of broilers on days 3, 13, 28, and 42. (**B**) Statistics of feed intake during the period of 3–13d, 3–28d, and 3–42d. (**C**) Statistics of feed conversion ratio during the period of 3–13d, 3–28d, and 3–42d. Different lowercase letters (a, b, c) represent significantly different means (*p* < 0.05).

The results showed that compared with the Con group, the body weight in the Pb group significantly increased on days 13, 28, and 42 and increased in the Ab group on day 28 (*p* < 0.05) (Figure 1A). Compared to the Ab group, the body weight of the broilers in the Pb group increased significantly on day 13 (*p* < 0.05) (Figure 1A). The feed intake of the broilers treated with antibiotics significantly decreased compared to the Con and Pb groups (Figure 1B) (*p* < 0.05). Compared to the Con group, the feed intake of the Pb group was significantly decreased during the period of 3–28d (*p* < 0.05). During the entire growth period, the feed conversion ratio was decreased in the Pb and Ab groups compared to the Con group (*p* < 0.05), and there was no significant difference between the Pb and Ab groups (Figure 1C).

### 3.2. Organ Indexes and Plasma Biochemical Indicators

The liver index of the Pb and Ab groups was significantly lower than that of the Con group on D21; the abdominal fat index of the Pb group was lower than the Ab group (*p* < 0.05). The liver and abdominal fat indexes of the Pb and Ab groups were significantly lower than that of the Con group on D42 (*p* < 0.05). However, there were no significant differences in the breast muscle and bursa of the Fabricius indexes of the yellow-feather broilers among the different groups (*p* > 0.05) (Table 3).

The results of plasma biochemical indicators of yellow-feather broilers are shown in Table 4. The plasma TG concentration was increased in the Pb group compared to the Con group on D21 (*p* < 0.05), and there was no significant difference in TG between the Pb and Ab groups (*p* > 0.05). The plasma ALB concentration was significantly higher in the Ab and Pb groups compared to the Con group (*p* < 0.05). The plasma glucose concentration was increased in the Ab group compared to the Con and Pb groups on D42 (*p* < 0.05). Moreover, there were no significant differences in the plasma concentrations of ALT, AST, CHOL, HDL, and LDL at all the phases among the three groups (*p* > 0.05).

### 3.3. Morphology of the Small Intestines

**Figure 2:**
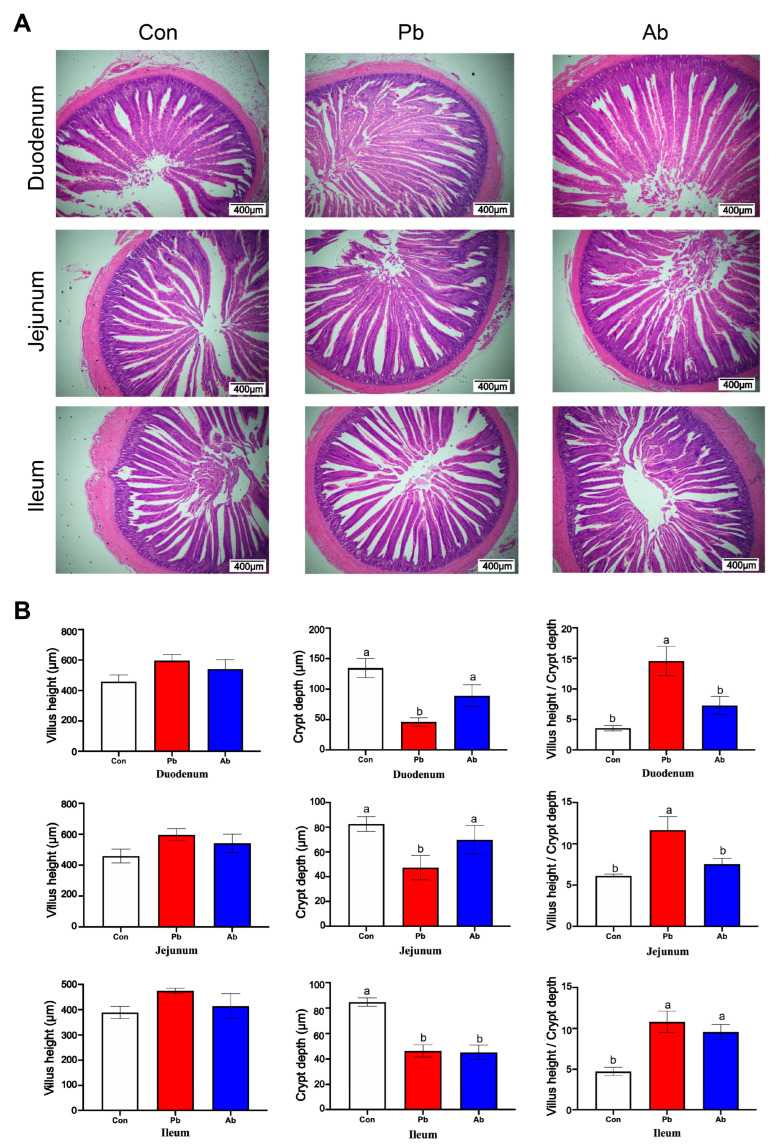
Changes in small intestinal morphology. (**A**) Representative HE staining sections of duodenum, jejunum, and ileum. (**B**) Statistics of villus length, crypt depth, and V/C value in the duodenum, jejunum, and ileum. Different lowercase letters (a, b) represent significantly different means (*p* < 0.05).

The histological structure of the small intestines is shown in Figure 2A. Compared with the Con group, the villus height did not show significant differences in duodenum, jejunum, and ileum in the Pb group (Figure 2B) (0.05 < *p* < 0.1). The probiotics treatment significantly reduced the crypt depth of the duodenum and jejunum compared to the Con and Ab groups (*p* < 0.05). The broilers in the Pb and Ab groups showed a decreased crypt depth in the ileum compared to the Con group (*p* < 0.05). The ratio of the villi height to the crypt depth (V/C value) in the duodenum and jejunum was significantly increased in the Pb group relative to the Con and Ab groups (*p* < 0.05); the V/C ratio of the ileum was significantly increased in the Pb and Ab groups relative to the Con group (*p* < 0.05).

### 3.4. Bacterial Community Characterization in Cecal Contents

**Figure 3:**
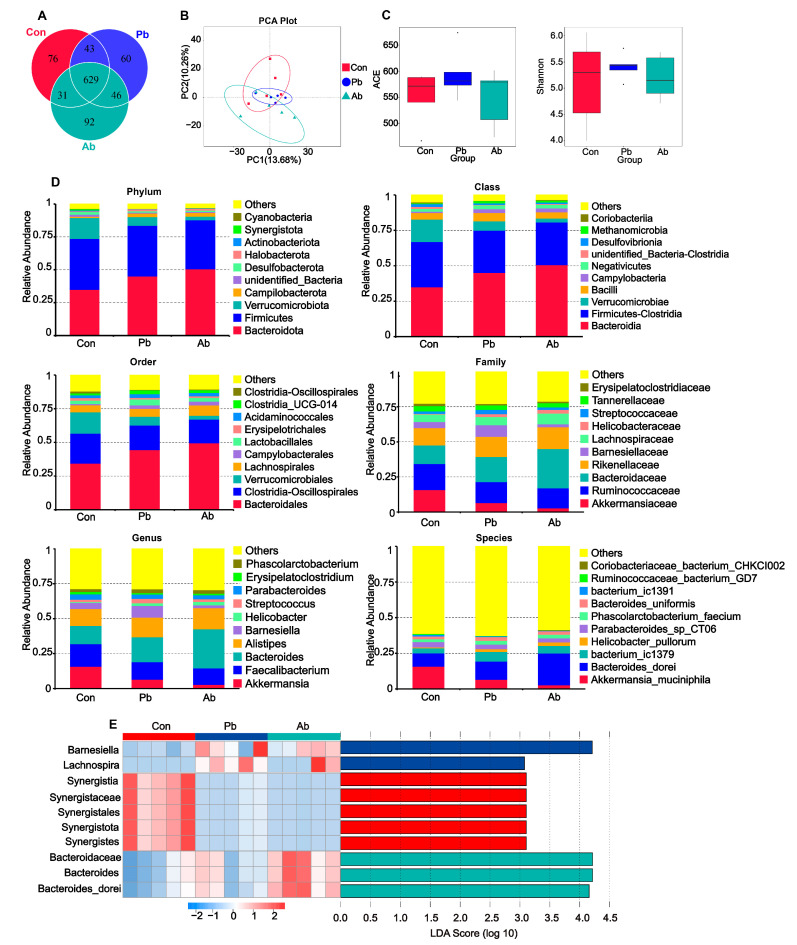
The Venn diagram and bacterial community composition of yellow-feather broilers fed with antibiotics or compound probiotics-supplemented diets at 42 d. (**A**) The Venn diagram summarizing the numbers of common and unique OTU in the microflora community in cecal contents of broilers. (**B**) The principal component analysis (PCA) plot about the cecal microbiota. (**C**) The ACE index reflecting species richness within and between groups. The Shannon index reflecting species diversity within and between groups. (**D**) Top 10 microbial composition in the cecum at the phylum, class, order, family, genus, and species levels. The other microbiota were combined into “others”. (**E**) The LEfSe analysis of cecal microbiota among treatment groups (a threshold value of >3 means, *p* < 0.05).

The Venn diagram showed that a total of 629 OTUs are shared among the three groups, and there were 60 and 92 unique OTUs in the Pb and Ab groups, respectively (Figure 3A). Although there were no significant differences in the ACE and Shannon indices among the groups (Figure 3C) (*p* > 0.05), the principal component analysis (PCA) analysis indicated that the microbiota structure in the Pb, Ab, and Con groups was different (Figure 3B). The unweighted pair group method with the arithmetic mean cluster tree revealed that the dominant bacterial phyla in the broiler cecal contents on D42 in our study were *Bacteroidota*, *Firmicutes*, and *Verrucomicrobiota* (Figure 3D). At the phylum level, the relative abundance of *Bacteroidota* was increased while *Verrucomicrobiota* was decreased in the Pb and Ab groups compared to the Con group. The abundance of Firmicutes in Pb was close to the Con group and showed slightly higher than that in the Ab group. At the class level, the abundance of *Bacteroidia*, *Fimicutes-Clostridia*, *Verrucomicrobiae*, and *Bacilli* was dominated; the relative abundance of *Verrucomicrobiae* in the Con group was more than that in the Pb and Ab groups. At the order level, the abundance of *Lactobacillales* in the Pb group was more than that in the Con and Ab groups. The dominant bacterial families in the cecal contents of the broilers were *Akkermansiaceae*, *Ruminococcaceae*, *Barnesiellaceae*, *Bacteroidaceae*, *Lachnospiraceae*, and *Rikenellaceae*. The relative abundance of *Barnesiellaceae* was increased in the Pb group, while *Bacteroidaceae* was increased in the Ab group compared to the Con group. Moreover, the relative abundance of *Streptococcaceae* in the Pb group was more than that in the Con and Ab groups. The relative abundance of the genus *Akkermansia* was higher in the Con group; the relative abundance of the genus *Bacteroides* increased in the Ab group; and the relative abundance of the genus *Barnesiella* and *Streptococcus* increased in the Pb group. At the genus level, *Bacteroides* and *Barnesiella were* significantly enriched in the Ab and Pb groups, respectively. At the species level, the relative abundance of *Bacteroides dorei* in the Ab group was higher than that in the Con and Pb groups. The bacterial differences were further studied with linear discriminant analysis (LDA), using the LDA effect size (LEfSe) algorithm (*p* < 0.05, LDA core > 3). The LDA effect size analysis showed that the microbiota with the highest absolute value of the LDA score in the Ab group was the family *Bacteroidaceae*, genus *Bacteroides*, and species *Bacteroides dorei* while, in the Pb group, it was *Barnesiella* (Figure 3E).

### 3.5. Functional Prediction of Microbiota

**Figure 4:**
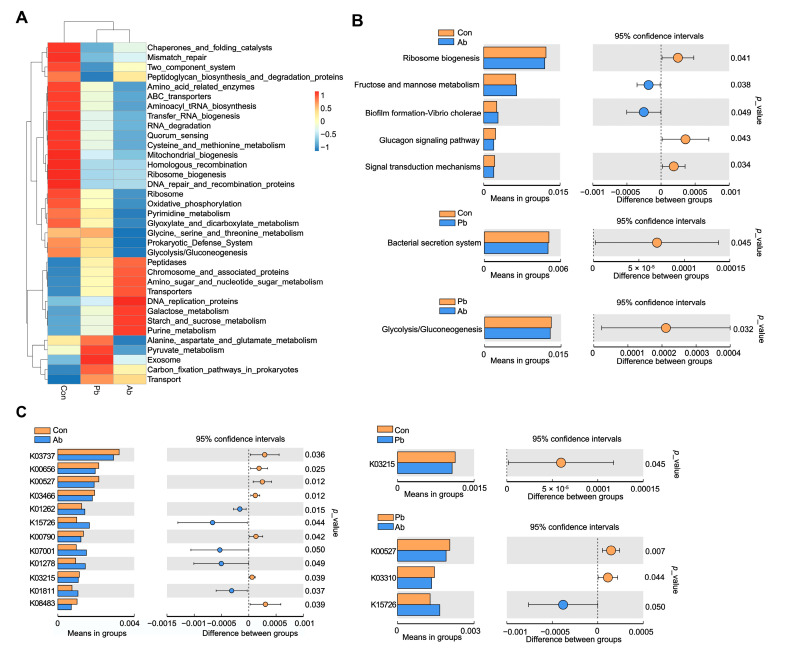
Predicted microbial function of cecal microbiota in broilers. (**A**) The heatmap of microbial functions predicted by Tax4Fun. (**B**) The comparisons of cecal microbial functions between treatments at Level 3 of KEGG pathways. (**C**) The comparisons of cecal microbial functions between treatments at Level K of KEGG pathways. Statistics were conducted by two-sided Welch’s t-test and Benjamini–Hochberg FDR correction between pairs of means, with *p*-value lower than 0.05 indicating a significant difference in microbial function.

As shown in Figure 4A, the heatmap showed the enrichment degree of metabolic pathways in Level 3 KEGG among all the groups. A total of seven metabolic pathways between the groups reached statistical significance (Figure 4B). Many of these metabolic pathways were related to carbohydrate metabolism and drug resistance, including fructose and mannose metabolism, glycolysis/gluconeogenesis, biofilm formation-vibrio cholera, and bacterial secretion system. The KEGG Orthology analysis showed 16 differential proteins (enzymes) between the groups (Figure 4C). Many of these proteins (enzymes) are involved in carbohydrate metabolism (K03737, K01278, K08483), amino acid metabolism (K03310, K01262), and drug resistance (K03215, K00527, K15726, K03737).

### 3.6. The Expression of Genes Related to Antibiotic Resistance

**Figure 5:**
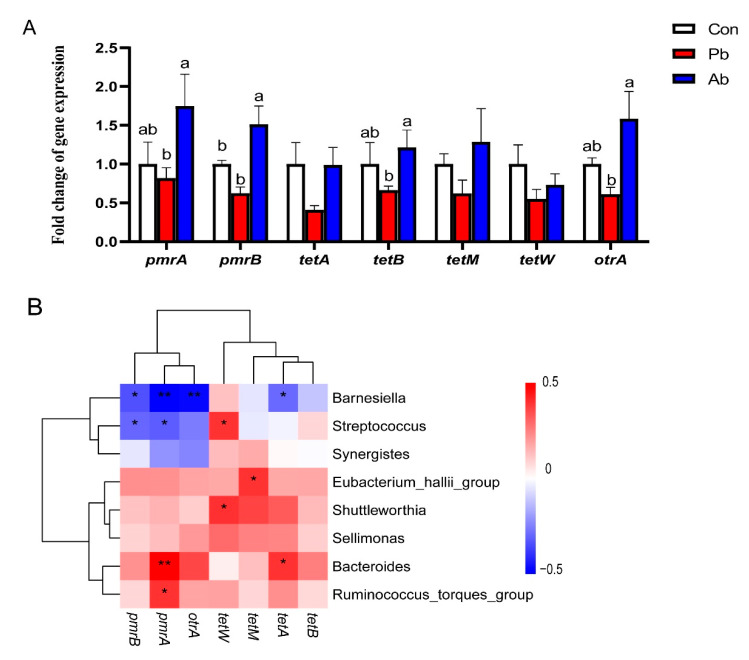
The expression of antibiotic resistance genes in cecal microbiota. (**A**) The expression of genes related to antibiotic resistance. Different lowercase letters (a, b) represent significantly different means (*p* < 0.05). (**B**) Spearman correlation analysis between cecal microbiota and antibiotic resistance genes (* *p* < 0.05, ** *p* < 0.01).

The qPCR results showed that the antibiotics treatment significantly promoted the expression of drug resistance genes, including *pmrA*, *pmrB*, *tetB*, and *otrA* compared to the probiotics-treated broilers, (*p* < 0.05); antibiotics significantly increased the expression of *pmrB* (*p* < 0.05) and mildly increased the expression of *pmrA*, *tetB*, and *otrA* compared to the Con group (0.05 < *p* < 0.1) (Figure 5A). In order to explore the internal relationships between the significantly different genera and drug-resistant genes, a Spearman correlation analysis was carried out. The results showed that *Barnesiella* was negatively correlated with the drug-resistant genes (*p* < 0.05), while *Bacteroides* was significantly positively correlated with *pmrA* and *tetA* expressions (*p* < 0.01) (Figure 5B).

### 3.7. The Level of Total Nitrogen, Total Phosphorus, and Ammonia Nitrogen in Feces

**Figure 6:**
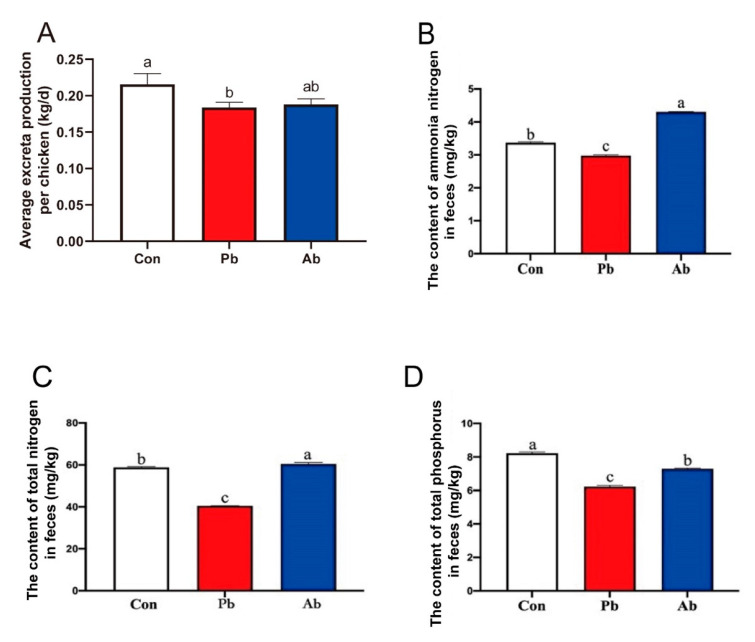
Excreta production and the contents of total nitrogen, total phosphorus, and ammonia nitrogen in feces. (**A**) Average excreta production per chicken (d 39–d 41). (**B**) The content of ammonia nitrogen in feces. (**C**) The content of nitrogen in feces. (**D**) The content of phosphorus in feces. Different lowercase letters (a, b, c) represent significantly different means (*p* < 0.05).

Daily fecal emissions were recorded continuously for 3 days before being slaughtered. The data showed that the probiotics treatment significantly reduced the average excreta production compared to the Con group (Figure 6A) (*p* < 0.05). The levels of total nitrogen and ammonia nitrogen in the feces were significantly increased in the Ab group relative to the Con and Pb groups (Figure 6B,C) (*p* < 0.05); the content of total nitrogen and ammonia nitrogen was significantly decreased in the Pb group compared to the Ab and Con groups (Figure 6B,C) (*p* < 0.05). Feeding the probiotics decreased the content of total phosphorus compared to the Con and Ab groups (Figure 6D) (*p* < 0.05).

## 4. Discussion

In recent years, probiotic preparations have been increasingly used in the poultry industry, and research on probiotics in improving poultry growth and health has become a hotspot. A meta-analysis of the effects of lactate-producing bacteria and yeasts as probiotics on the growth performance of broilers showed that probiotics can increase body weight and body weight gain, and reduce the feed conversion ratio [17], which is highly consistent with our data. An important reason for adding antibiotics to feed is due to the growth-promoting effect. In this study, the growth-promoting effect of compound probiotics on broilers is higher than that of antibiotics, indicating that replacing antibiotics with probiotics has a great prospect in broiler breeding.

Excessive abdominal fat deposition not only threatens the health of broilers but decreases meat quality and economic value [18]. In this study, the abdominal fat index of the Pb group was lower than that of the Con and Ab groups. Moreover, a significant increase in the plasma glucose level in the Ab group suggested that antibiotics may cause metabolic disorders in broilers. Previous studies showed that antibiotic treatment can cause disorders of multiple metabolic pathways in chickens [19]. The small intestine is the main part of material digestion and nutrient absorption. The normal structure and function of the small intestine is the basic guarantee of sufficient digestion and absorption of nutrient substances [20]. The intestinal villus length and crypt depth are important indexes to evaluate the digestion–absorption function of the small intestine. The higher the villi height the better the intestinal digestion and absorption function [21]. These results were consistent with the study by Qiu et al. [22], which revealed an improved intestinal tissue morphological structure by adding probiotics to broilers’ diets. A profitable effect of probiotics is to promote the development of the intestinal epithelium. Supplemented with *L. acidophilus* in the intestinal mucous membrane induced the differentiation of the intestinal microvillus through the target signal pathway [23]. Then, the signal was transported into the base of the intestinal mucous membrane and stimulated the differentiation of the intestinal stem cell to induce the development of the villus [23]. Therefore, probiotics increase the nutritional utilization function of the small intestine, which may be an important reason for the improvement of growth performance in broilers.

Gut microbiota play important roles in regulating host digestive functions, immune response, and metabolic regulation [24]. Diet is an important factor leading to altered intestinal microbiome, a complex ecosystem with a dynamic diversity of species [25]. Our Venn diagram and PCA analysis indicated that the composition of the cecal microbiota was changed by the treatment with antibiotics and probiotics. The relative abundance of *Lactobacillales*, *Streptococcaceae*, and *Streptococcus* was increased in the Pb group, indicating the probiotics were well colonized in the gut. Further analysis showed that, at the genus level, *Barnesiella* and *Bacteroides*, the dominant bacteria, were increased significantly in the Pb and Ab groups, respectively. At the species level, *Bacteroides dorei*, which can induce intestinal inflammation and have long been considered bacterial pathogens [26], were significantly increased in the Ab group.

The application of antimicrobials results in the emergence and spread of antimicrobial resistance; the addition of antibiotics in the poultry feed undoubtedly exacerbated the adverse effects [27]. It is well documented that the use of antibiotics in poultry production increases the selection pressure for antibiotic-resistant bacteria [28]. Moreover, *Bacteroides* that significantly increased in the Ab group also act as a reservoir of resistance determinants. *Bacteroides* isolates, dwelling as seemingly innocuous members, can serve as reservoirs of resistance determinants, which they can pass on to much more virulent bacteria [29,30]. Therefore, in this study, the expression of the antibiotic resistance genes was detected in the cecal microbiota. The *pmrA*-*pmrB* are associated with colistin resistance; a previous study reported that the activated transcription of *pmrA*-*pmrB* contributed to the antimicrobial peptide resistance of *Salmonella typhimurium* [31]. Active efflux and ribosome protection are the main mechanisms of tetracycline resistance; *tetA* and *tetB* participate in active efflux, whereas *tetM*, *tetW*, and *otrA* participate in ribosome protection [32]. Our data showed that the antibiotics treatment significantly upregulated the expression of *pmrA*, *pmrA*, *tetB*, *and otrA* compared to the Con group; however, the probiotics treatment significantly reduced this gene expression compared with the Ab group. The Spearman correlation analysis showed that the genera *Barnesiella*, which significantly increased in the Pb group, was negatively correlated with *pmrB*, *pmrA*, *otrA*, and *tetA*. Previous studies demonstrated that *Barnesiella* can eliminate intestinal pathogens and modulate immune responses [33,34]. Ubeda *et al.* found that, in a murine model, the restoration of *Barnesiella spp*. following fecal microbiota transplantation was associated with the elimination of the vancomycin-resistant enterococci [34]. These findings indicate that an increased abundance of *Barnesiella* contributes to reduced antibiotic resistance in broilers. Although there is no case of direct use of *Barnesiella* as a probiotic in broilers, there is a possible symbiotic relationship between *Barnesiella* and other probiotics, like *Lactobacillus* and *Bacillus*, in broilers. Zhu et al. found that the basal diet plus compound probiotics (*Bacillus subtilis* and *Lactobacillus acidophilus*) significantly increased the abundance of *Barnesiella* in cecal contents [35]. Therefore, using compound probiotics to indirectly promote the growth of *Barnesiella* may also be an effective pathway. In addition, the function prediction of microbiota showed that K15726, which is named as a heavy metal efflux system protein in KEGG, significantly increased in the Ab group. A previous study reported that heavy metal exposure is one of the factors causing the antibiotic resistance of pathogens [36]. The heavy metal efflux system protein may provide a direction to reduce drug resistance in broilers.

In this study, the function prediction of microbiota also showed that many microbial functions affected by antibiotics and probiotics were associated with amino acid metabolism. Although the small intestine is the main site of amino acid absorption, the role of the caeca in amino acid metabolism cannot be ignored. A previous study showed that significant amounts of nondigested dietary amino acids flowed into the caeca and then were subsequently metabolized by the cecal microbiota [37]. Therefore, we speculate that the altered cecal microbial function may affect the emission of nitrogen in feces. Consistently, compared to the Con and Ab groups, the contents of the total nitrogen and ammonia nitrogen were significantly reduced in the Pb group. These results were consistent with the findings of Wei et al., who reported that reduced ammonia production in the blood by the dietary supplementation of probiotics might be due to the degradation of ammonia by *Bacillus subtilis* [38]. Abd El-Hack et al. found that, in addition to reducing nitrogen, supplementation with *Bacillus subtilis* can also decrease excreted phosphorous in laying hens [39]. Our data also confirmed that compared with the Con and Ab groups, probiotic treatment significantly reduced the total phosphorus in feces. It is well known that the nitrogen, ammonia, and phosphorus in feces are the main cause of water eutrophication. Therefore, decreased nitrogen, ammonia, and phosphorus, as well as excreta production, indicated that the altered cecal microbiome induced by probiotics can improve production efficiency within environmentally friendly conditions.

## 5. Conclusions

This present study compared the effects of probiotics and antibiotics supplementation on the growth performance, cecal microbiome, and fecal emission of broilers. Our results revealed that although supplementation with compound probiotics or antibiotics can decrease the feed conversion ratio, the probiotics treatment had more advantages. Compared to the other groups, probiotics significantly improved the morphological structure in the small intestine. The 16S rRNA sequencing analysis showed that the genus *Barnesiella* and *Bacteroides* were the most significantly enriched bacteria in the probiotic and antibiotic treatment groups, respectively. The functional prediction of the microbiota showed that the probiotic and antibiotic treatments had different effects on drug resistance, carbohydrate metabolism, and amino acid metabolism. The correlation analysis further confirmed that *Baenesiella* negatively correlated with drug resistance genes (*pmrB*, *pmrA*, *otrA*, and *tetA*), while *Bacteroides* positively correlated with drug resistance genes (*pmrA* and *tetA*). In addition, the probiotic treatment reduced the emission of nitrogen and phosphorus in feces, while antibiotics did not have this effect. In summary, probiotics can completely replace feed antibiotics for the broiler industry and have significant advantages in improving intestinal histological structure, gut microbiota composition, drug resistance, and pollution emissions.

## Figures and Tables

**Table 1:** The ingredients in the diets and the nutritional composition.

	Ages (Day)	
Items	1–21	22–42
Ingredients (%)		
Corn	56.90	59.66
Soybean meal	33.00	32.20
Fish meal	3.00	0
Limestone	1.20	1.15
Dicalcium phosphate	1.45	1.65
Soybean oil	3.00	3.90
L-methionine	0.15	0.14
Sodium chloride	0.30	0.30
Vitamin-mineral premix *	1	1
Nutritional composition (%)		
Crude protein	20.98	18.83
Total lysine	1.17	1.00
Total methionine and cysteine	0.80	0.72
Calcium	1.08	0.88
Nonphytate phosphorus	0.45	0.40
Total phosphorus	0.68	0.66
Metabolic energy (Mcal/kg)	2.96	3.00

* Supplied per kilogram of diet: vitamin A, 7000 IU; vitamin D_3_, 2500 IU; vitamin E, 30 mg; vitamin K_3_, 2 mg; vitamin B_1_, 3 mg; vitamin B_2_, 5 mg; pantothenic acid, 800 mg; choline chloride, 1500 mg; nicotinic acid, 30 mg; pyridoxine, 3 mg; folic acid, 500 mg; biotin, 0.2 mg; vitamin B_12_, 1 mg; Fe,100 mg; Cu, 8 mg; Mn, 100 mg; Zn, 100 mg; I, 0.42 mg; and Se, 0.3 mg.

**Table 2:** Primer sequences of the target genes.

Gene	Primer Sequences (5′-3′)
*pmrA*	F: AGTTTTCCTCATTCGCGACCA
	R: TACCAGGCTGCGGATGATATTCT
*pmrB*	F: GGATGGCCTGATGTGACGCTGTC
	R: GCGCGGCTTTGGCTATATGCTG
*tetA*	F: TTGGCATTCTGCATTCACTC
	R: GTATAGCTTGCCGGAAGTCG
*tetB*	F: AGTGCGCTTTGGATGCTGTA
	R: AGCCCCAGTAGCTCCTGTGA
*tetM*	F: ACAGAAAGCTTATTATATAAC
	R: TGGCGTGTCTATGATGTTCAC
*tetW*	F: GAGAGCCTGCTATATGCCAGC
	R: GGGCGTATCCACAATGTTAAC
*otrA*	F: GGCATYCTGGCCCACGT
	R: CCCGGGGTGTCGTASAGG

**Table 3:** Effect of dietary supplementation with compound probiotics or antibiotics on the organ index in yellow-feather broilers.

Items, %		Con	Pb	Ab
Liver	D21	2.59 ± 0.12	2.64 ± 0.10	2.57 ± 0.14
	D42	2.79 ± 0.35 ^b^	1.95 ± 0.05 ^a^	1.91 ± 0.06 ^a^
Breast muscle	D21	4.58 ± 0.11	4.89 ± 0.07	4.90 ± 0.13
	D42	8.37 ± 0.21	9.49 ± 0.56	9.21 ± 0.35
Abdominal fat	D21	1.17 ± 0.09 ^ab^	0.96 ± 0.03 ^a^	1.33 ± 0.86 ^b^
	D42	2.56 ± 0.10 ^b^	1.54 ± 0.13 ^a^	1.80 ± 0.12 ^a^
Bursa of Fabricius	D21	0.24 ± 0.03	0.23 ± 0.03	0.26 ± 0.02
	D42	0.24 ± 0.03	0.23 ± 0.02	0.21 ± 0.01

Con, basal diet; Pb, basal diet supplemented with 100 mg compound probiotics; Ab, basal diet supplemented with 16.5 mg/kg zinc bacitracin and 3.3 mg/kg colistin sulfate; and results are shown as mean ± standard error (n = 6). ^a,b^ means in the same row with different superscripts differ (*p* < 0.05).

**Table 4:** Effect of dietary supplementation with compound probiotics or antibiotics on the blood biochemical parameters in yellow-feather broilers.

Items		Con	Pb	Ab
ALT	D21	1.67 ± 0.88	1.67 ± 0.67	3.17 ± 1.01
	D42	3.38 ± 0.84	2.75 ± 0.37	2.14 ± 0.14
AST	D21	205.67 ± 6.15	233.67 ± 10.67	225.50 ± 9.31
	D42	223.88 ± 16.42	228.25 ± 8.53	218.00 ± 6.82
GLU	D21	13.19 ± 0.38	12.37 ± 0.27	13.26 ± 0.36
	D42	12.20 ± 0.25 ^a^	12.03 ± 0.24 ^a^	15.34 ± 1.52 ^b^
TG	D21	0.47 ± 0.03 ^a^	0.60 ± 0.02 ^b^	0.57 ± 0.06 ^ab^
	D42	0.54 ± 0.03	0.45 ± 0.03	0.49 ± 0.05
CHOL	D21	3.40 ± 0.15	3.70 ± 0.14	3.70 ± 0.18
	D42	3.70 ± 0.10	3.69 ± 0.08	3.85 ± 0.09
HDL	D21	2.60 ± 0.12	2.82 ± 0.08	2.92 ± 0.12
	D42	2.29 ± 0.08	2.45 ± 0.07	2.47 ± 0.09
LDL	D21	0.53 ± 0.06	0.71 ± 0.60	0.66 ± 0.09
	D42	0.91 ± 0.12	0.74 ± 0.04	0.80 ± 0.08
ALB	D21	12.87 ± 0.27 ^a^	13.88 ± 0.41 ^b^	14.17 ± 0.08 ^b^
	D42	13.44 ± 0.47	13.79 ± 0.21	13.77 ± 0.24

Con, basal diet; Pb, basal diet supplemented with 100 mg compound probiotics; Ab, basal diet supplemented with 16.5 mg/kg zinc bacitracin and 3.3 mg/kg colistin sulfate; ALT, alanine transaminase; AST, aspartate transaminase; GLU, glucose; TG, triglyceride; CHOL, cholesterol; HDL, high- density lipoprotein; LDL, low-density lipoprotein; and ALB, albumin. Results are shown as mean ± standard error (n = 6). ^a,b^ means in the same row with different superscripts differ (*p* < 0.05).

## Data Availability

The data presented in this study are available on request from the corresponding author. The data are not publicly available due to privacy.
